# Trauma, adversity, and biological aging: behavioral mechanisms relevant to treatment and theory

**DOI:** 10.1038/s41398-024-03004-9

**Published:** 2024-07-12

**Authors:** Kyle J. Bourassa, David A. Sbarra

**Affiliations:** 1 VA Mid-Atlantic Mental Illness Research, Education and Clinical Center, Durham VA Health Care System, Durham, NC USA; 2 Geriatric Research, Education, and Clinical Center, Durham Veteran Affairs (VA) Health Care System, Durham, NC USA; 3 Center for the Study of Aging and Human Development, Duke University Medical Center, Durham, NC USA; 4 Department of Psychology, University of Arizona, Tucson, AZ USA

**Keywords:** Human behaviour, Biomarkers, Genetics

## Abstract

Although stress and adversity are largely universal experiences, people exposed to greater hardship are at increased risk for negative health consequences. Recent studies identify accelerated biological aging as a mechanism that could explain how trauma and adversity gives rise to poor health, and advances in this area of study coincide with technological innovations in the measurement of biological aging, particularly epigenetic profiles consistent with accelerated aging derived from DNA methylation. In this review, we provide an overview of the current literature examining how adversity might accelerate biological aging, with a specific focus on social and health behaviors. The most extensive evidence in this area suggests that health-compromising behaviors, particularly smoking, may partially explain the association between adversity and accelerated aging. Although there is relatively less published support for the role of social behaviors, emerging evidence points to the importance of social connection as a mechanism for future study. Our review highlights the need to determine the extent to which the associations from adversity to accelerated aging are consistent with causal processes. As we consider these questions, the review emphasizes methodological approaches from the causal inference literature that can help deepen our understanding of how stress and trauma might result in poor health. The use of these methodologies will help provide evidence as to which behavioral interventions might slow aging and improve health, particularly among populations that more often experience adversity and trauma.

## Introduction

In the 87 years since Hans Selye first integrated the concept of stress into the study of health [1–3], a wealth of empirical research now demonstrates that people who experience stress and adversity are at greater risk of illness and premature death [4–7]. Findings in this area link a wide array of stress and adversity exposures with numerous health outcomes that span disease states, organ systems, and the human lifespan [8, 9]. For example, compared to people who report less stress, people under greater stress are more likely to develop respiratory illnesses when exposed to the same viruses [10]. Similarly, people with greater stress show slower healing than less stressed people with the same wounds [11–13]. At a population health level, widowed adults are twice as likely to die during the week following their spouse’s death and almost three times as likely to develop heart disease [14, 15]. Despite convincing evidence that these associations are robust, identifying the biological processes that explain how adversity impacts the body has proven challenging. What psychological and behavioral processes activate or maintain disease-relevant physiology among people exposed to high levels of stress? Over the last decade, emerging empirical evidence points to biological aging as a multisystem physiological mechanism that could help explain how adversity translates to ill health, and the latest work in this area builds on critical stress-health associations observed over the last century.

Advances in the study of biological aging have coincided with a developing area of research focused on the study of health and aging, termed geroscience [16–18]. A key theme within geroscience is that aging is the strongest correlate of chronic disease and premature death [19]. In this framework, slowing aging—specifically the aging of the body, termed biological aging—has the potential to reduce the risk for disease and death across multiple organ systems, reducing disability and improving longevity as people grow older chronologically [19–23]. The value of biological aging as a mechanism linking stress to health is, in part, due its disease-agnostic nature, which better matches myriad published associations between stress and disease. This review summarizes the current literature linking stress, trauma, and adversity to biological aging, with a specific focus on behaviors that might explain this association, specifically social and health behaviors. We conclude by highlighting the limitations of the current literature linking adversity to biological aging and opportunities to move this area of research toward stronger causal inference that can ultimately support future theoretical and intervention advances.

## The many metrics of biological aging

Biological aging refers to the progressive deterioration of physiological systems and functions that are required for survival [24]. Chronological age—that is, chronological time since birth—is not the same as biological age, and a major theme in modern geroscience is in identifying and studying heterogeneity in aging profiles and health decline [25–27]. Because many chronic diseases—e.g., cardiovascular disease, metabolic diseases, neoplastic diseases, and Alzheimer’s disease—are more common as people age chronologically, the search for markers of accelerated biological aging has garnered considerable scientific attention [28]. Work in this area includes but is not limited to, assessing changes in genomic stability and DNA methylation (DNAm), cell senescence, and mitochondrial functionality [28]. Telomeres, proteins protecting chromosome ends [29, 30], were among the earliest biomarkers of aging, and telomere length plays an important role in cellular senescence [31], with the shortening of telomeres reflecting accelerated biological aging. Leukocyte telomere length is associated with all-cause mortality [32], risk for cardiovascular disease [33], and risk for Alzheimer’s disease [34]. Importantly, psychosocial stressors are believed to play a role in accelerating telomere shortening via sympathoadrenal, neuroendocrine, and/or immune-inflammatory mechanisms that heighten oxidative stress and damage telomere ends [35]. Other metrics have proven useful in operationalizing biological aging, including the development of the pace of aging [36] and other equations [37, 38] that use biomarkers spanning multiple organ systems to characterize the coordinated deterioration of physiological function. For example, Belsky et al. [36] developed, refined [39], and validated a pace of aging metric in the Dunedin Study birth cohort across 19 biomarkers. They showed that the pace of aging for individuals in this cohort—all born in the same year and thus the chronological age since birth—was associated with cognitive and physical health decline in midlife.

As geroscience became increasingly interested in the dissociation between chronological and biological aging, technical advances in molecular sequencing technologies [27], especially the quantification of epigenetic DNAm, have led to the emergence of a series of methylation clocks [40, 41]. In brief, the extant clocks and methylation-based pace of aging metrics are based on DNAm of cytosines at CpG dinucleotides, which has direct relevance for RNA gene expression [28, 42]. Changes in epigenetic mechanisms are considered a hallmark of aging biology [17], though given the use of machine learning approaches to derive methylation measure algorithms, it is unclear whether epigenetic measures of aging are directly accessing biological aging or indirect, but useful, biological correlates [43].

Although it is beyond the scope of this review to detail all of the available epigenetic clocks (interested readers can see recent reviews, [41, 44]), we can summarize key trends in the literature as follows: (1) Following a series of seminal papers in 2013 [45, 46], several DNAm clocks emerged in the literature, many of which were trained to predict chronological age [27]; (2) Although many of the different clock metrics are correlated with each other and other aspects of biological aging, these correlations are often low and suggest that each clock measures different aspects of biological aging [44]. The first generation metrics, born from machine learning algorithms designed to predict chronological age, do not have clear links to the biology that underpins aging, which led to the development of second-generation clocks designed to more fully capture the functional changes associated with biological aging [47], some of which are specifically designed to predict mortality risk [48–50]. Most recently, epigenetic measures of aging have emerged that were directly trained on longitudinally assessed measures of biological aging, such as DunedinPACE [42], resulting in a third-generation of clocks—or perhaps a new category of DNAm measure altogether. These DNAm measures are increasingly used to predict mortality risk and healthspan [42, 51]. The ability to assess biological aging at a single point in time using DNAm, which would typically require costly and time-consuming longitudinal assessment, has allowed for a host of new social and behavioral research linking biopsychosocial predictors to health via accelerated aging.

## The many dimensions of adversity and associations with accelerated aging

Adversity can be conceptualized a number of ways, spanning the type, timing, and duration of a stressful experience. The measurement of stress and adversity can include the assessment of exposure to stressful events or situations, as well as people’s subjective experiences of adversity, typically by measuring their perceptions of stress. Stressful events are measured in a variety of ways. One common method is counting the number of stressful experiences an individual has experienced, such as the number of adverse childhood experiences (ACEs; [52, 53]) or stressful life events [54–56]. More recently, there have been calls for integrative and detailed assessment of stressful events [57] to better capture the range and timing of stressors beyond counts [58, 59]. Despite measurement challenges, ACEs (abuse, neglect, and household dysfunction experienced by children) and stressful life event counts are linked to accelerated aging [55, 60, 61]. For example, experiencing common stressful life events is associated with changes in biological aging among adolescents [62]. However, stress constructs that assess specific events should not be considered exhaustive. Other common adversities linked to biological aging include growing up in socioeconomic disadvantage [63, 64], membership in a racial or ethnic minority group [65, 66], and discrimination [67]. For example, African American adolescents who had higher levels of perceived discrimination showered faster biological aging if they did not have a supportive family environment [68]. These broader measures of chronic adversity almost certainly play an important role in modulating the experiences of stress and aging across the lifespan.

A subset of stressful events—i.e., traumas, criterion A traumas, or traumatic events—are defined as particularly harmful and have the potential to cause posttraumatic stress disorder, a collection of symptoms related to re-experiencing traumatic events, avoiding reminders of trauma, and experiencing changes in cognition, mood, and arousal. The hyperarousal symptoms that contribute to the diagnosis of PTSD correlated with dysregulations in physiology, including changes to cardiovascular physiology and inflammation [69–72] and poor health. More recent work finds that the experience of trauma [73–75], as well as the development of PTSD [75, 76], are linked to accelerated aging. For example, in a sample of 339 trauma-exposed veterans, higher levels of PTSD hyperarousal symptoms were associated with high DNAm biological age [77]. Notably, associations of trauma and PTSD with accelerated aging span multiple methods of assessment. People with PTSD show accelerated aging as assessed by functional tests [78], telomeres [79], pace of aging [55], and epigenetic measures of aging (i.e., DNAm; [71, 73]).

The experience of stress and trauma can be contrasted with measures of adversity that seek to capture people’s subjective experiences of stress. The same event experienced by different people can have a different impact on health depending on individual characteristics, including genetic vulnerability to stress, personality, or availability of psychological services. Widely used measures like the Perceived Stress Scale [80] seek to capture people’s subjective feeling of stress and show consistent associations with biological aging [55, 81–84]. Stress perceptions are typically self-reported and have the advantage of being cost-effective and simple to administer. Notably, both stressful event counts and subjective perceptions of stress can vary across developmental periods with different relevance to aging. There is a necessary component of timing and duration implicit within the experience of adversity or stressful events, both in terms of acuity versus chronicity [85], and the specific timing within human development (e.g., spanning infancy, childhood, adulthood, midlife, and older age). Different rates of aging and epigenetic outcomes have been linked to adversity across many periods of the human lifespan [86–88], including slowed prenatal development of the central nervous system [89, 90], accelerated aging in childhood [86], and different rates of biological aging among older adults [91].

## Behavioral mechanisms linking adversity to accelerated aging

**Fig. 1:**
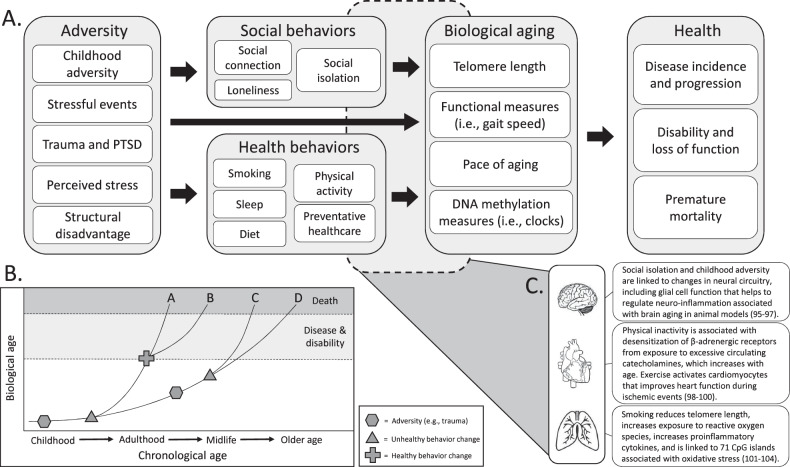
Figure outlining links between adversity, social/health behavior, accelerated aging, and health. **A** Conceptual pathway linking adversity to health through changes in health behaviors, social behaviors, and subsequent changes in biological aging. **B** Illustration of how adversity could influence the rate at which a person ages biologically by increasing the likelihood of unhealthy behavior change, which accelerates biological aging and hastens progressions towards, disease, disability, and death. **C** Specific illustrative examples of molecular mechanisms shown to link health behaviors to biological aging in the brain, heart, and lungs.

It is widely known that early adversity and childhood stress are associated with poor distal health outcomes [92, 93], as is the experience of chronic psychological stress [8]. Although it is certainly the case that the experience of stress and trauma can have direct, dysregulatory effect on physiological systems in a manner that accelerates biological aging (cf. [94–104]), it is also likely that intermediate behaviors play a role in the causal chain from adversity to accelerated aging and poor health. Figure 1 provides a process model framework for understanding the ways in which stress, adversity, and trauma may portend behavioral changes that accelerate biological aging. Although it is possible to prevent some trauma and adversity from occurring or to reduce stress responses through treatment, it is impossible to prevent all stress and adversity. Identifying potentially health-relevant sequelae of adversity that could accelerate aging would present additional mechanistic targets for mitigating the adverse consequences of the stressful experiences. Here we present current evidence for two broad classes of potential mechanisms of action: social connectedness and health behaviors. Following Fig. 1, our review of the extant evidence on these topics is framed in terms of what we know about (a) the associations between adversity, stress, trauma and behavioral mechanisms, and (b) the associations between social/health behaviors and accelerated biological aging. We posit that changes in these potential mediating processes play a role in sculpting physiological responses that accelerate biological aging. In short, stress, adversity, and PTSD often alter our social relationships and health behaviors. High stress may have causal effects on social withdrawal and/or portend relationship conflict; it may disrupt sleep, and people may smoke to manage the adverse emotional experiences of the stress itself. In turn, these behaviors organize changes in physiological functioning that may alter genomic signaling in a way that contributions to illness and disease progression.

### Social connection and disconnection

Social connectedness is a multifactorial umbrella term that represents the degree to which people are and perceive themselves to be embedded in nurturing social relationships [105]. Included under the social connectedness umbrella are structural elements of social network properties (e.g., isolation or living alone) and social roles (e.g., marital status), functional components (including perceived social isolation, which is the definition of loneliness), and the quality of these close relationships (see [106]). The available evidence indicates that adversity, stress, and trauma are highly correlated with disrupted social relationship functioning in adulthood. Early social adversity plays a critical role in shaping infant and adult attachment orientations, which organize the mental models people hold about the reliability and trustworthiness of others in close relationships [107, 108]. Childhood maltreatment is a significant risk factor for the perpetration of intimate partner violence [109]. Socioeconomic adversity in childhood is a strong predictor of relationship dissolution in young adulthood, with evidence indicating early socioeconomic adversity predicts relationship dissolution directly as well as indirectly through interpersonal conflict and low levels of future orientation [110]. The available evidence also suggests that PTSD is highly associated with poor relationship functioning and increased risk for divorce [111–113], and this appears to be especially true for combat-exposed veteran populations [114]. In addition, meta-analytic and experimental evidence has indicated that PTSD both predicts and is predicted by low levels of social support [115, 116]. For example, Bourassa and colleagues found that exposure therapy increased perceived social support among active duty solders with PTSD in an experimental trial [116]. Finally, recent evidence suggests that, at the level of the US population, loneliness increased during the COVID-19 pandemic in relation to pre-pandemic levels [117], and these disturbances in social functioning are hypothesized to play a role in shaping pandemic-related increases in mental health disorder [118].

Although the associations between adversity, stress, trauma and social relationship functioning are well established, the literature on social relationship functioning and accelerated biological aging is relatively nascent. When compared to married adults, those who are separated or divorced evidence shorter salivary and leukocyte telomere length [119, 120]. Interestingly, telomere shortening does not appear to mediate the elevated risk between marital status and cardiovascular disease mortality [119]. Relatively new research from the Health and Retirement Study (HRS) suggests that perceived social support from and contact with close others, especially close friends, were *prospectively* associated with slower pace of aging (assessed via DunedinPoAm38) and lower GrimAge, two indices of epigenetic (DNAm) age acceleration [121]. Independent analyses with the HRS data provide a conceptual replication of these findings and find that the absence of marital and friend relationships are related to an older GrimAge and faster pace of aging [122]. The inability to establish peer relationships characterized by autonomy and relatedness in adolescence is also associated with older GrimAge at age 30 [123], and these variables accounted for nearly 8% of the variance in epigenetic age acceleration over and above variance accounted for by cigarette smoking and demographic factors. In a unique, but small (*N* = 40), study of work-related trauma exposures among paramedicine students, GrimAge acceleration was significantly lower among participants who reported increased social support at baseline and follow-up [124]. In a longitudinal study of African American adolescents in rural Georgia, Brody and colleagues [66] found that high family support moderated the relationship between experiencing racial discrimination and epigenetic age acceleration (at ages 20 and 22 years). Adolescents from high support families who experienced high and stable discrimination showed comparable age acceleration to people not experiencing discrimination, whereas those adolescents experiencing discrimination and having a low support family showed up to 2 years of epigenetic age acceleration on a first-generation DNAm clock. A family-based intervention research with the same cohort showed reductions in harsh parenting in the treatment condition was associated with reduced epigenetic age acceleration [67]. This finding, in particular, supports the hypothesis that social relationship functioning can serve as a critical mechanism linking life stress and biological age acceleration.

### Health behaviors

Health behaviors present some of the most plausible mechanisms that could explain how adversity accelerates biological aging, with extensive empirical support for the associations between adversity, unhealthy behaviors, and accelerated aging. Evidence for these associations exist across an array different adversities, unhealthy behaviors, and measures of aging across the lifespan. For example, childhood adversity is linked to unhealthy behaviors in midlife [125], as well as to smoking, physical inactivity, risky sexual behavior, problematic alcohol use, and problematic drug use in a meta-analytic framework [126]. The association between adversity and unhealthy behavior extends to later periods of life as well. Adults who report experiencing more stressful life events or higher levels of perceived stress are more likely to also smoke [127–129], have poor diet [130, 131], or be physically inactive [132, 133]. Older adults who experience interpersonal stressors, such as divorce or widowhood, are also more likely to be physically inactive or smoke [134–136]. Adversity is associated with less engagement with health protective behaviors as well—people exposed to more stress and adversity are more likely to endorse vaccine hesitancy [137] and may delay preventative healthcare [138, 139]. The broad associations between adversity and unhealthy behaviors also extend to trauma and PTSD. People with PTSD are at greater risk of smoking, physical inactivity, poor diet [140], and engaging in risky behaviors [140–142], though there is some evidence that these associations are explained by depressive symptoms [143]. The health behavior of sleep is notable as a possible link between PTSD and accelerated aging. Sleep disturbance is one of the central features of PTSD [144] and is associated with both PTSD [145–147] and stress, broadly speaking [148]. Notably, PTSD treatment improves sleep outcomes [149], suggesting an element of reversibility of this risk with efficacious PTSD treatment.

**Fig. 2:**
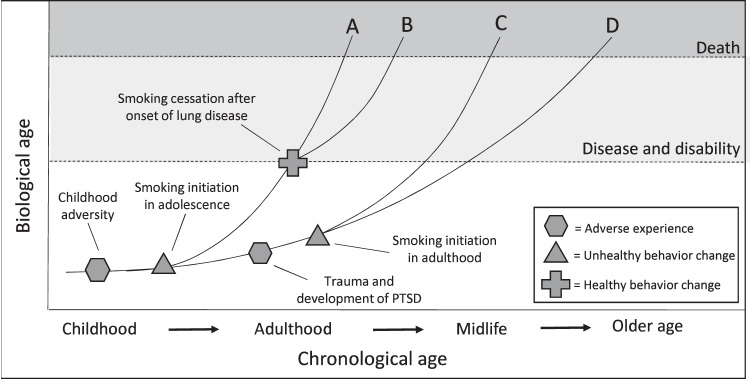
Illustrative example of how childhood adversity and traumatic stress could accelerate biological aging by increasing risk for smoking across the lifespan, as well as how intervening to promote smoking cessation might help slow aging after people experience adversity. Paths that diverge at behavior change points (i.e., paths A–D) represent different theoretical rates of aging for different smoking behaviors (i.e., smoking initiation or not, smoking cessation or not) that could emerge among people who experience adversity.

Health behaviors are perhaps the most robust predictors of biological aging, with the expectation that unhealthy behavior leads to poor health through accelerated aging, at least in part. Accelerated aging is associated with heavy alcohol use [150, 151], cannabis use [152], diet [153–157], physical activity [153, 158], and disturbed sleep [159]. Summarizing the extensive literature linking biological aging and health behaviors—even if limited to epigenetic measures of biological aging—is beyond the scope of this review. However, two points are notable. First, the associations between health behaviors and epigenetic measures of aging have initial evidence of reversibility. For example, Waziry and colleagues [156] found that caloric restriction was experimentally associated with slowed epigenetic aging in the CALERIE study, though the degree to which these changes are associated with improved long-term health is unknown. Evidence that changes in health behavior result in changes in aging measures would represent gold-standard evidence as to the most promising targets for future interventions attempting to slow aging among people experiencing adversity. Second, there is an extensive body of empirical evidence linking health behaviors to accelerated aging focused on smoking [50, 102–104, 160–163]. This includes evidence of broad associations [103, 160, 161], as well as changes in tissue specific to the lungs [102, 162]. For example, a recent study using the Health and Retirement Study found that lifetime history of exposure to cigarette smoke was associated with multiple epigenetic measures of aging, which helped explain the link between smoking and the development of chronic diseases and premature mortality [163]. Indeed, the association between smoking and biological aging is so well established that it is common for researchers to control for smoking status in studies or even in the creation of methylation clocks themselves. For example, methylation-derived pack years are included in the GrimAge clock’s calculation [50], due to the clear association between smoking and mortality risk. This creates a conundrum when studying stress, adversity, and accelerated aging; namely, smoking is likely both on the causal pathway linking adversity to aging [129], directly associated with changes in methylation (Fig. 1), and in some cases, included as part of the outcome being assessed [50]. Figure 2 illustrates how biological aging could change over time based on changes in smoking behavior related to adversity experienced across the lifespan. It is notable, however, that several studies find that indicators of stress and adversity, including PTSD [74, 76], remain associated with accelerated aging when controlling for smoking, suggesting that while smoking might help explain the association between adversity and aging, there are likely other mechanisms of action that require future study.

## Incorporating the science of causal inference into the study of adversity and accelerated aging

Although the associations between early adversity, psychological stress, trauma, and accelerated biological aging are well established, much of the work in this area is correlational; indeed, the same can be said for most of the research on the putative social and health behavior “mechanisms” that link these constructs. One of the main limitations—and areas of opportunity—in the study of adversity and aging is the lack of more rigorous studies that can speak to causal processes. Experimental studies and randomized controlled trials, of course, remain the gold standard for assessing the causality of adversity and associations with age acceleration (or any other health, health-relevant, or disease biomarker). As we discuss below, interventions can target the psychological consequences of adversity, or they can directly target the mechanisms that link life stress exposures to accelerated biological aging (see Fig. 1). With relatively few exceptions [67], however, this research has yet to be conducted in humans. Nevertheless, there exists a promising suite of tools for making better correlational inferences when relying on observational data [164–170], and our contention is that research on adversity and accelerated biological aging will benefit enormously by using these tools (cf., [171]). Table 1 includes several examples of the methods and potential applications to questions of causal inference in the study of adversity and accelerated biological aging.

**Table 1:** Illustrative listing of causal inference methodologies that could support deeper study of the association between adversity, stress, trauma, and accelerated biological aging.

Method	Basic description	Illustrative examples for the study of accelerated biological aging^a^
Directed acyclic graphs (DAGs)	DAGs provide a visual illustration of causal assumptions and assist in identifying variables that need statistical control and situations where controlling for specific variables may worsen causal inferences [196].	A DAG representation of the potential confounding influences from early life adversity to accelerated aging in midlife is used to identify *collider variables* that artificially strengthen the association.
Propensity score analyses (PSA)	In some situations, stress exposures are random; in other situations, especially in cases of adversity, life event exposures are non-random. Propensity scores allow researchers to condition on a set of covariates that are used to predict exposure to the traumatic experience. The resulting propensity scores can then be used as a means of matching exposed vs. non-exposed groups, or by weighting participants in terms of their likelihood of being exposed to a traumatic experience [170, 197].	In a longitudinal study of combat-exposed veterans, propensity scores are used to examine the association between PTSD diagnoses and accelerated aging. Study participants are matched on their likelihood to experience PTSD. The results show that without propensity score matching (PSM), PTSD is associated with accelerated epigenetic aging. After PSM, the association between PSTD and accelerated epigenetic aging is not reliably different from zero.
Instrumental variable (IV) analysis	IV analysis is a method that helps account for unmeasured confounding in observational analyses. (Thus, whereas PSA methods account for measured confounders, IV methods account for unmeasured confounders.) IV estimation is based on a series of assumptions, the chief of which is that the instrument variable associated with the exposure is not associated with unmeasured confounds (see [198]). Increasingly, genetically-informed Mendelian randomization methods are used in health sciences as a form of IV analysis [199].	A group of investigators use Mendelian randomization methods to study the bidirectional association between PTSD and a methylation-based pace of aging outcome. The results reveal significant associations from the pace of aging to PSTD but not the reverse, which raises questions about the directionality of extant associations in the literature.
Natural experiments	Natural experiments occur when an event or condition affects a specific sub-population, creating a situation that is analogous to a randomized experiment and thus addresses many of the confounds inherent observational research [200]. There are many potential natural experiments in the stress-health literature, but the key is to ensure that the stress exposure is indeed random—e.g., comparing counties exposed to chronic stress of environmental contamination may seem like a natural experiment, but the question of whether such contaminations are random is open to debate.	To the extent that government policies, environmental emergencies, or crises vary randomly, a study can address whether people living in counties or cities exposed to such stressors (vs. those who are unexposed) show accelerated biological aging. In many situations, the naturally-occurring event can be considered an instrumental variable—e.g., see [201] for a study of the Vietnam lottery draft as a means of estimating the causal effect of veteran status. In this case, the participants are drafted randomly and the lottery status is an IV for veteran status.
Target trial emulation studies	Based on counterfactual theory, target trial emulation (TTE) methods attempt to recreate the conditions of a randomized clinical trial within observational data [202]. A key step in the TTE method is matching eligibility criteria within an observational data set, then following these people through some type of exposure to a pragmatic trial.	TTEs involve [1] identifying a causal question from observational data in the form of a hypothesized RCT protocol, and [2] emulating the components of the protocol. For example, in a longitudinal research study of perceived stress, match participants in a manner consistent with an RCT protocol, then evaluate if changes in perceive stress are associated with changes in accelerated aging (see [203] for details on implementation).
Sibling-comparison designs	Among a suite of quasi-causal within-family models, sibling-comparison designs offer a power test of causal life event exposure and other environmentally-mediated hypotheses [204]. Genes and environments are highly confounded—e.g., as a stress exposure, divorce is non-random, and thus studying its putative consequences (e.g., in the association between divorce exposure and biological aging) cannot rule-out the confounding role of genetics. Sibling-difference analyses represent a form of random genetic assignment during meiosis and can be a useful tool in assessing the environmental impact of within-family differences in stress exposures.	In a large longitudinal study that includes full biological siblings, researchers investigate whether exposure to chronic interpersonal stress and strain is associated with accelerated biological aging. The sibling comparison model involves creating a within-family average of interpersonal stress and strain, then centering each sibling’s score on this metric relative to the family-level average. The main hypothesis centers on the extent to which siblings with elevated stress and strain scores (relative the family average) show more accelerated epigenetic aging.
Cotwin control designs	Among a suite of quasi-causal within-family models, cotwin control designs provide another method that helps account for genetic confounding [191]. Cotwin control designs take advantage of the fact the monozygotic (MZ) twins share identical genotypes and when compared with dizygotic (DZ) twins, who share only 50% of their genetic material, this yields a biometric model that can help rule out genetic confounding in stress exposure to health outcome associations [192].	In a sample of MZ and DZ twin pairs, researchers study whether differential exposure to high levels of subjective psychological stress is associated with prospective changes in accelerated biological aging. Within this design, researchers examine whether, after accounting for genetic influences in the stress-aging association, within twin-pair differences in chronic psychological stress remains a significant predictor of accelerated biological aging.
G-Computation	The nonparametric g(general) formula for estimating causal effects has many applications and is based on estimating the probability of an outcome under different hypothesized conditions based on different sets of control variables [170, 172, 205]. G-computation is based on counterfactual theory and the conditional probabilities of the outcome under different exposure conditions.	Researchers are interested in the potential age acceleration consequences of caregiving stress, and outcome groups are created to classify participants into groups of people who do and do not show epigenetic age acceleration. Covariates include gender and a history of adverse childhood experiences. G-computation is completed on the marginal means to compare the age acceleration of those exposed vs. unexposed to caregiving stress under the different control conditions. The difference of these average probabilities is then calculated to create an estimate of the causal effect of caregiving on age acceleration.

^a^These examples are not drawn from the empirical literature; instead, they were created to illustrate the potential application of the methods outlined in this paper.

Two core themes in this literature are relevant to the current review. First, confounding is endemic in observational research and cannot be (fully) addressed by covariate adjustment alone [172–174]. Central to this theme is the idea that life event exposures are non-random, and any effort to understand the putative consequences of early adversity, mid-life discrimination, or combat-related trauma must contend with the individual differences and demographic variables. These individual characteristics almost certainly play a role in shaping who is exposed and how often they are exposed to stressful events, the impact of these stressors on behavior and functioning, and the potential biological age acceleration alleged to result from adversity. Second, all statistical methods make certain assumptions and these assumptions limit causal inference conclusions that can be drawn in any area of study. Consequently, researchers are increasingly calling for the *triangulation* of methodological and statistical approaches when dealing with questions of causal inference [175, 176]. For example, recent research on the effects of smoking on suicidal ideation and attempts compared the results of between-family analyses to those following Mendelian randomization (MR) analyses, which is an effective method for evaluating residual confounding and potential reverse causation [177]; importantly, the observational/correlational analyses showed a strong positive association between the constructs, but the quasi-causal MR analyses showed no evidence for a causal effect. (It is important to keep in mind that any interpretation of these findings depends on the quality of the MR analysis. As a general conceptual framework, triangulation depends on the strength of the data that is informing the causal inference strategy. In other words, efforts toward triangulation depend not only on the assumptions of all the triangulating approaches (a broad feature of this work), but also the quality of the data available in a particular study (a specific limitation in the application of the method).

It is common for different methodological approaches to yield dissociated findings (and this is especially true when research incorporates analyses from between- and within-family models, see [168, 178, 179]) and triangulating across different approaches is a key element of scientific falsification [180, 181], the powerful idea that scientific advances unfold not through the slow accumulation of evidence in favor of an association of interest, but instead the inability to rule-out the plausibility of the association. Statistical methods that make different assumptions can provide increased opportunities for falsification, which can increase confidence in the robustness of a result. Alternatively, a lack of consensus across methods and the accumulation of discrepant findings during triangulation can provide the initial evidence that a single scientific paradigm is insufficient for solving the scientific question at hand [182], leading to new discoveries that challenge the status quo. When combined with modern causal inference analytic procedures (see Table 1), triangulation is a powerful conceptual tool for identifying causal effects. Importantly, the methods outlined in Table 1 are complementary—alone, none have the power to rule-out a causal effect and each method has a set of biases and assumptions; but when used together via triangulating approaches, especially in cases where RCTs are not possible, these methods are powerful.

What methods might be most helpful in moving the study of adversity and accelerated biological aging toward greater confidence in causal processes? We detail three approaches with the potential to help advance our understanding of this topic: RCTs targeting mechanisms of action, longitudinal mediational analyses, and twin studies.

### Randomized controlled trials (RCTs) targeting mechanisms of action

The NIH Science of Behavior Change (SOBC; https://commonfund.nih.gov/behaviorchange) initiative was designed to move intervention science from a broad implementation of treatment packages (for health behavior change) to a focus on targeted mechanisms of action [183, 184]. Key components of this work involve showing that (a) interventions can engage those targets (referred to as *target engagement*), (b) that changes in these mechanistic targets yield changes in the outcomes of interest (referred to as *target validation*)—terms with origins from pharmaceutical development and the broad field of experimental medicine (see [185]). Related to the topic of adversity, trauma and accelerated aging, the goal of experimental research would be showing that targeting social relationship functioning or health behaviors could then slow biological aging. For example, among combat-exposed veterans with diagnosed PTSD, can cognitive behavioral therapy for insomnia (CBTi) improve sleep and are improvements in sleep quality or duration associated with differences in accelerated aging? These interventions might be bundled with other treatment strategies, including those that focus on social functioning and/or medication, within the context of factorial experimental designs [186]. As the gold standard, increased use of RCTs seems a promising next step in the use of epigenetic aging measures.

Despite the promise of RCTs, it is important to note that using epigenetic aging outcomes in geroprotective trials has limitations, including questions about the reliability of measurement and periods required to observe meaningful changes in biological aging. Current epigenetic measures show variability in reliability of measurement that would make RCTs more challenging. For example, Higgins-Chen and colleagues reported that using principal-component (PC) adjusted clocks results in around 1.5 years of deviations between replicates [187]. Although an improvement over the 9 years found in non-PC adjusted clocks, this level of variability remains a barrier to detecting therapeutic effects. Similarly, the ideal timeline to detect clinically- or statistically-relevant change in epigenetic measures of aging is unclear, and any measurement strategy must be timed to capture the precise resolution of the causal change process [188]. For clock metrics trained on mortality, multiple years might be necessary to detect reliable change, limiting the value of RCTs that follow participants for under a year. Measures trained on physiological aging (e.g., DunedinPACE), might produce reliable change over a shorter period, however, this remains to be tested directly. These challenges are not unique to RCTs, but would make these studies challenging to conduct successfully given the cost and time involved in clinical trials. However, there is the potential that new clocks or PC approaches might produce measures that are more reliable in the future. If RCTs routinely collect methylation data, it would allow for future reanalysis of trials. Regardless of the ultimate method, linking changes in social and/or health behavior to changes in biological aging through RCTs or some other form of experimental intervention study would be critical to support theory and future intervention research aiming to slow biological aging.

### Pursue longitudinal mediational studies

Longitudinal mediation refers to models that help interrogate the extent to which intervening variables, termed mediators, explain the observed association between a predictor and outcome [189]. The most rigorous mediation models use multiple assessments of the relevant variables and can provide evidence as to the causal pathways that might link a broad risk factor, such as adversity, with aging or health outcomes via putative mechanisms of action. For example, a longitudinal cohort that assessed PTSD, perceived social support, and epigenetic aging at multiple occasions would allow researchers test whether people who develop PTSD evidence decreases in their perceived social support, which in turn predict accelerations in epigenetic measures of aging. Such approaches are particularly valuable in modeling change in mediators and outcomes that help overcome issues of temporal precedence and the directionality of associations. Although some studies are beginning to examine changes in aging at multiple occasions, much of the recent biological aging research has been limited to cross-sectional associations. As more longitudinal epigenetic data becomes available in longitudinal cohorts and laboratory studies, conducting more mediation analysis become feasible. For example, Wolf and colleagues recently found that GrimAge mediated the association between externalizing psychopathology and inflammatory phenotypes in a sample of 214 trauma-exposed military veterans [190]. Mediation analyses that examine whether a putative casual factor that explains the associations between exposure and outcome can serve as a valuable first step in determining future intervention targets. The use of longitudinal mediation models, particularly with multiple assessments of aging, health behavior, and social connection, would provide more stringent evidence as to how adversity might affect aging, albeit with limited ability to make specific causal claims.

### Take advantage of cotwin control designs

**Fig. 3:**
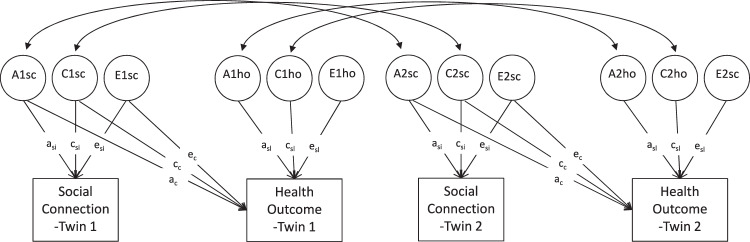
Illustration of co-twin control model (bivariate Cholesky). The critical test of interest is the extent to which differences in social connection (sc) within a twin pair predict differences in a health outcome (ho) after accounting for genetic and shared environmental influences common to both variables.

RCTs and longitudinal mediation are highly time and resource intensive study designs. As we note above, one of the challenges that emerges outside of RCTs is confounding; for example, it is quite possible that neuroticism is associated with both stress, adversity, and trauma exposures, as well as poor sleep and increased likelihood of smoking, all of which are associated with accelerated biological aging. Cotwin control designs [191, 192] are uniquely suited to examine genetic confounding. Applied to the example above, genetic confounding would operate when the genetics of smoking, for example, account for the phenotypic association between smoking and accelerated aging. Cotwin designs leverage the genetic relatedness of twins to create a counterfactual situation [193]. Because monozygotic twins share a genotype, a biometric decomposition that compares twins who are and are not exposed to a specific life event (or, who are differ quantitatively in their responses to life event exposures) allows research to control for genetic and shared environmental influences that may confound the exposure/outcome association. In this sense, cotwin designs provide a powerful falsification methodology that operates in many ways akin to a hierarchical regression analysis: After accounting for shared genetic and environmental influences between a putative exposure and outcome, do within-pair differences in twins’ exposure remain significantly associated with the outcome in question [191]? Figure 3 provides an illustration of a biometric cotwin model examining whether (within-pair) differences in stress exposure or life adversity are associated with metrics of accelerated biological aging (see Table 1 as well). This model specification allows us to examine whether an intrapair difference in a predictor variable (including, for example, discordance in PTSD diagnoses or quantitative differences in perceived stress) with an intrapair difference in a metric of epigenetic age acceleration. If the observed (i.e., phenotypic) association between the intrapair differences in X and Y remains significant after accounting for genetic and shared environmental confounds, the findings are consistent with a causal relationship and the estimated effect size of the association will be less biased because two major sources of confounding are attenuated/removed [191].

## Using causal inference to advance theory and treatment

How would the use of casual inference advance our knowledge of how stress, trauma, and adversity might accelerate biological aging? The value of causal inference is tied to the aims of the research program in question. Most studies of biological aging and adversity present statistical associations without the ability to make causal claims, however the objective of these findings are generally focused on one of two long-term targets— (1) better understanding the causal pathway that might link adversity to accelerated aging and poor health, i.e., theory, or (2) clinical interventions that are attempting to slow aging and improve health for people affected by high levels of stress, adversity, or trauma, i.e., prevention and treatment. Said differently, some programs of research aim to “carve nature at its joints,” whereas others are more agnostic about the reasons for causality and instead are focused on slowing aging in clinical settings. In the case of explicating the potential causal pathway from adversity to poor health, studies that are limited to associations, and particularly cross-sectional associations, cannot move the field towards causal inference. As stated above, statistical controls and temporal ordering can help limit alternative explanations for observed associations, but there are limits to how close observation studies can cleave to causal inference. Similarly, animal studies have an important role to play in the study of adversity and aging [193, 194], though drawing inferences from animal models comes with its own challenges [194, 195]. As a result, it is critical to move beyond findings that show broad associations between adversity and biological aging in humans if we wish to determine how adversity might cause more rapid aging and downstream health outcomes. Without using more sophisticated methods, describing the causal pathways from stress to health will remain challenging.

In the case of supporting interventions aiming to slow aging to improve health, the use of causal inference might appear less critical. Any association between stress and aging, or putative mediators, presents a plausible intervention target that might then be tested using RCTs as the gold standard methods of causality. The translational pathway—from observing broad risk, to evidence of mediation for plausible mechanisms, to testing interventions in an RCT framework—is defensible in the abstract and might achieve the stated goal of determining how to slow aging. However, it is notable that relatively few prospective RCTs have examined whether interventions slow aging generally (for an example, see the CALERIE trial, [156]), and fewer still among people who have experienced adversity or trauma. Although this may reflect the nascent nature of the area of research in this area, which might change as epigenetic measures of aging present a more realistic surrogate endpoint for use in RCTs, the cost and difficulty with running RCTs will remain, particularly given concerns with reliability and timeline when assessing epigenetic measures of aging [187]. Mechanisms of slowing aging that have evidence more closely approaching causal inference would presumably have a higher likelihood of a successful trial. Such evidence could be more convincing to the stakeholders necessary to fund and conduct aging interventions, including granting agencies, medical system administrators, and the clinicians delivering the interventions.

## Conclusions and future directions

In this review, we have briefly summarized the state of the literature linking adversity to accelerated aging, including two behavioral mechanisms that might explain this association, social connection and health behaviors. Given the existing literature, there is a need to use rigorous methodologies to move observational studies stress and aging closer towards causal inference. The use of RCTS, longitudinal mediation models, and cotwin control designs, for example, would help provide more confidence that putative mechanisms—such as social and health behaviors—explain how adversity is linked to accelerated aging. This evidence would provide a stronger theoretical understanding of the causal pathways from adversity to accelerated aging and poor health, which would support future intervention efforts to slow aging and improve health among people who experience stress, trauma, and PTSD.
